# The Effect of a Video-Assisted Health Education Program Followed by Peer Education on the Health Literacy of COVID-19 and Other Infectious Diseases Among School Children: Quasi-Randomized Controlled Trial

**DOI:** 10.2196/43943

**Published:** 2024-01-29

**Authors:** Xiaojuan Zhang, Yingkun Justin Wen, Ning Han, Yawen Jiang

**Affiliations:** 1 School of Public Health (Shenzhen) Sun Yat-sen University Shenzhen, Guangdong China; 2 School of Public Health Sun Yat-sen University Guangzhou China; 3 Department of Learning, Informatics, Management and Ethics Karolinska Institutet Stockholm Sweden; 4 Institute of Public Health Supervision of Longgang District Shenzhen China

**Keywords:** infectious diseases, primary school students, quasi-randomized controlled trial, video-assisted health education, peer education, item response theory, IRT

## Abstract

**Background:**

To improve the engagement and effectiveness of traditional health programs, it is necessary to explore alternative models of health education including video-assisted lectures and peer education.

**Objective:**

This study aimed to evaluate the effects of a combination of video-assisted lectures and peer education on health literacy related to infectious diseases among students.

**Methods:**

Third-grade classes from 11 pilot schools in Longgang District of Shenzhen, China, were randomized to the intervention and control groups. In the intervention group, a video-assisted interactive health education program was conducted twice over a time span of 5 months. Each of the 2 sessions included a 40-minute lecture on COVID-19 and other common infectious diseases in schools and a 5-minute science video. In addition, 5 “little health supervisors” at the end of the first session were elected in each class, who were responsible for helping class members to learn health knowledge and develop good hygiene habits. Students answered the same quiz before the first and after the second session. Models based on item response theory (IRT) were constructed to score the students’ knowledge of infectious diseases based on the quiz.

**Results:**

In total, 52 classes and 2526 students (intervention group: n=1311; control group: n=1215) were enrolled. Responses of the baseline survey were available for 2177 (86.2%; intervention group: n=1306; control group: n=871) students and those of the postintervention survey were available for 1862 (73.7%; intervention group: n=1187; control group: n=675). There were significant cross-group differences in the rates of correctly answering questions about influenza symptoms, transmission, and preventive measures; chicken pox symptoms; norovirus diarrhea symptoms; mumps symptoms; and COVID-19 symptoms. Average IRT scores of questions related to infectious diseases in the intervention and control groups were, respectively, –0.0375 (SD 0.7784) and 0.0477 (SD 0.7481) before the intervention (*P=*.01), suggesting better baseline knowledge in the control group. After the intervention, the average scores of the intervention and control groups were 0.0543 (SD 0.7569) and –0.1115 (SD 0.7307), respectively (*P*<.001), suggesting not only significantly better scores but also greater improvement in the intervention group.

**Conclusions:**

After the health education project, the correct answer rate of infectious disease questions in the intervention group was higher than that of the control group, which indicates significant effects of the combination of video-assisted lectures and peer education for the promotion of health literacy. In addition, the intervention effect of the first session persisted for at least 4 months up to the second session. As such, the proposed program was effective in improving the health literacy of school children in relation to infectious diseases and should be considered for massive health promotion campaigns during pandemics.

**Trial Registration:**

ISRCTN ISRCTN49297995; https://www.isrctn.com/ISRCTN49297995

## Introduction

Primary school students are vulnerable to emerging and common infectious diseases such as COVID-19, influenza, mumps, and intestinal infectious diseases [1]. A survey on the reasons for sick leaves in primary and secondary schools in Shenzhen showed that the top 5 causes were common cold, gastrointestinal diseases, unexplained or other illness, influenza, and chicken pox [2]. In addition, the importance of acquiring essential knowledge regarding the prevention and control of COVID-19 cannot be overstated during the pandemic. Accordingly, it is critical to embed health promotion into the school education of primary school students. To that end, the outline of “Healthy China 2030” emphasizes the importance of fortifying health education among school children. In particular, primary schools were integral to the life cycle of the health education curriculum to the extent that early-life exposure to information on diseases and health behaviors is associated with improved future health outcomes [3].

Despite its importance, health education was highly restricted in its delivery forms. Conventionally, the most prevalent approach of health education of infectious diseases for school students was, arguably, classroom lectures aided with paper-based materials, in which the teaching contents are usually compiled by school teachers and researchers [4]. Traditional health education is also reported to have a limited duration of effects. Hampered by the collective challenges faced in traditional health education, most schools lack systematic health education programs [5]. To increase students’ interest in healthy behaviors and to extend the duration of education effects, researchers have been exploring alternative media for health education. Among the various new models, two of the prevailing approaches are video-assisted health education and interactional peer education [5,6].

In professional medical education, video-assisted lectures are useful tools for students to acquire basic clinical skills. When delivered in bundle with in-person lectures, video-based materials are often preferred by students [7]. In addition, video-assisted health education has been shown to be more effective than oral education in facilitating postoperative recovery of patients [8].

The effects of health education are not necessarily limited to the immediate recipients of the program themselves. Students may also help to shape the opinions and behaviors of their classmates by becoming peer educators of health and hygiene. Peer education is defined as “sharing experiences and learning among people with something in common,” such as a similar age, living environment, and culture [9]. There is substantial evidence that peer education is highly effective in specific areas of medical and health education, including professional medical training, chronic disease prevention, and sexual health behaviors [6,10,11]. Incorporating peer effects into the design of health education programs could, therefore, strengthen the programs' impacts on behavioral change.

However, evidence on the effects of video-assisted lectures and peer education on health literacy among school children is still lacking. Given its substantial potential for public health practice, we designed a health education package that combined video-assisted classroom teaching and peer education and tested the effectiveness of this program. This program, which we anecdotally refer to as the “Little Health Supervisors” project, was anticipated to improve the health literacy of students over an array of infectious diseases.

## Methods

### Trial Design

The “Little Health Supervisors” project is jointly enacted by the Longgang District Bureau of Health and the Longgang District Bureau of Education as an administrative task. Third-grade classes from 11 pilot schools in Longgang district of Shenzhen, China, were randomized to the intervention and control groups. Our aim was to allocate equal numbers of third-grade classrooms to the intervention and control groups within each school. However, schools with an odd total number of classes inevitably resulted in uneven groups; hence, one group might outnumber another eventually. This project enclosed 2 health education sessions 4 months apart in Dec 2021 and Apr 2022 in Longgang District, Shenzhen City in the Guangdong Province of China, which is a district with approximately 4 million residents and 0.4 million school students.

### Ethical Considerations

The “Little Health Supervisors” project was launched by the district government as a public service project. The study protocol was approved by the Biomedical Research Ethics Review Committee, School of Public Health (Shenzhen), Sun Yat-sen University [2021(056)] and was registered with Longgang District Bureau of Health (Figure S1 in Multimedia Appendix 1). Informed consent was obtained from all students and their parents who met the inclusion criteria and were willing to participate. Confidentiality of information was maintained.

### Recruitment

**Figure 1:**
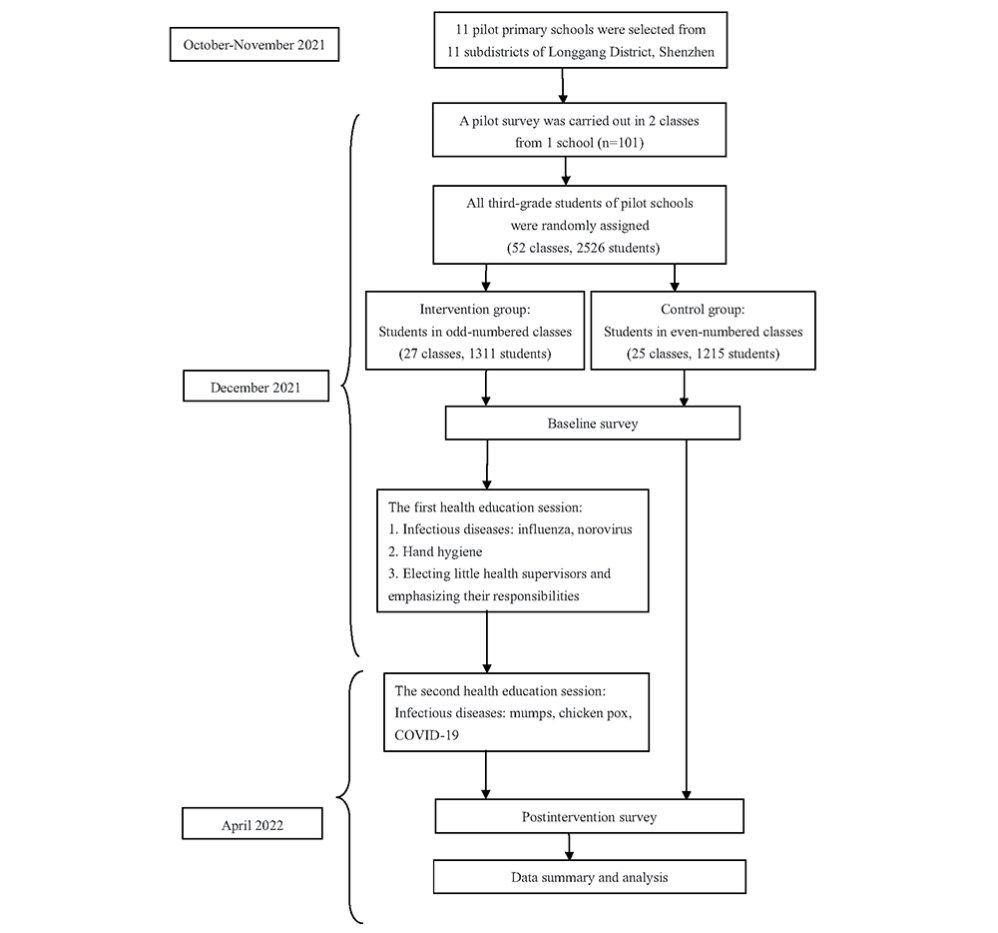
Flowchart of the "Little Health Supervisors" project (a cluster randomized controlled trial) from December 2021 to April 2022.

In the first step of sample enrollment, considering the feasibility of the project’s implementation, the Longgang District Bureau of Health and the Longgang District Bureau of Education recommended 1 primary school based on the willingness to participate for each of the 11 subdistricts of this district. Second, all third-grade students in the 11 schools were eligible for participation if they met the following requirements: (1) they were not taking a leave of absence from school at the time of enrollment; (2) they agreed (or their guardians agreed) to spend time on attending lectures; (3) they had access to a computer, tablet, or smartphone with an internet connection; (4) they had sufficient knowledge to use mobile devices or computers (assistance allowed); and (5) they were able to read and interpret Chinese characters. Next, as decided by the researchers, the eligible students were assigned to the intervention and control groups using the class number as the randomizer. Specifically, odd-numbered classes were assigned to the intervention group; even-numbered classes, the control group. The flowchart of participant enrollment is illustrated in Figure 1.

### Data Collection

To standardize students’ knowledge of infectious diseases both before and after the education program, a questionnaire containing a quiz on COVID-19 and selected infectious diseases with relatively high local incidences was curated, which included influenza, chicken pox, norovirus diarrhea, and mumps. The questionnaire also collected demographic characteristics (school, class, student number, sex, and date of birth) and COVID-19 vaccination status. In addition, we delivered a separate questionnaire to a parent of each student who collected parental assent to vaccinate their children against COVID-19. Moreover, family socioeconomic information was also collected in the parent questionnaire, which included monthly household income and the parents’ education level [12].

Questionnaires were distributed via a web-based survey platform (Wenjuanxing, Changsha Ranxing Information Technology Co, Ltd). In the baseline survey, students completed the questionnaires in a computer laboratory with the instructions of either the computer teachers or the class advisors. To collect parents’ responses, the teachers arranged a meeting with each family using previously connected social media to select a representative for questionnaire responses. Due to COVID-19 outbreaks during the planned time period of the second session, the postintervention survey was distributed on the web. Simultaneously, the researchers also collected the questionnaire from the control group.

### Interventions

The intervention was developed by both researchers and the local health department. Details of the development process and the content of the intervention are provided in Table S1 and Figures S2-S3 in Multimedia Appendix 1 [13,14]. Students randomized to the intervention group had access to 2 free sessions of health education during the study. Each session included a 40-minute lecture on the transmission and prevention of different infectious diseases, followed by a 5-minute science video. To incentivize learning, students were informed that there would be interactional question-and-answer sections during the lecture, for which the participating students were eligible for prizes.

In December 2021, the baseline survey and the first health education session were conducted, with the former preceding the latter. The in-person lecture and the videos of the first session pertained to influenza, norovirus diarrhea, and hand hygiene. At the end of this session, 5 little health supervisors were elected by the teachers from each class. They were naturally assumed as opinion leaders, showcasing their ability to effectively convey knowledge and could supervise the learning of health knowledge and the development of good hygiene habits of their classmates. The teachers also handed out brochures, armbands, and stickers to the 5 little health supervisors. In addition, the teachers encouraged all students to take health knowledge home and improve the family's health literacy by way of “small hands holding big hands,” which aimed to exploit the power of two-step flow theory of communication for information transmission. Originating from political science, the two-step flow theory asserts that information can be conveyed through the chain of media-opinion leaders-audience. Students may also help to shape the opinions and behaviors of their family members by becoming an opinion leader of health and hygiene [15,16].

In April 2022, the second health education session and the postintervention survey were carried out. However, the order of education and survey was reversed in relation to the first session. The lecture and the videos of the second session pertained to chicken pox, mumps, and COVID-19 symptoms. Affected by a local COVID-19 outbreak, students had to take the web-based classes at home, so the health education sessions had to be conducted in the form of recorded course videos. In the intervention group, students were required to watch the video, and the teachers also encouraged all students to distribute health knowledge to the people around them.

As for the control group, the students only received routine health education at school, which included health tips on influenza from school doctors and 1 or 2 public welfare courses conducted by the local health department or hospitals every semester. These routine health education sessions were balanced between the 2 groups.

### Outcomes

The primary outcomes of this trial were the score in the original scale (hereafter referred to as “crude score”) and item response theory (IRT) score of questions related to infectious diseases, the correct answer rates of questions related to infectious diseases, and the pre-post changes in the correct answer rates after the intervention. The secondary outcomes were the COVID-19 vaccination rates. For those who did not receive COVID-19 vaccines at baseline or at the end of the program, we also exploratively asked about their willingness to get vaccinated and the reasons for not being vaccinated.

### Statistical Analysis

To gain an overview of students’ characteristics, their families’ demographic data were collected. Monthly household income (in ¥) was categorized into 4 levels (<¥5000 [US $702.97], ¥5000 [US $702.97]~¥10,000 [US $1405.94], ¥10,000 [US $1405.94]~¥20,000 [US $2811.88], and ≥¥20,000 [US $2811.88]). Parent’s education was grouped into 3 levels (junior high or below, secondary school [including technical secondary school], and college and above]. For the questions related to infectious diseases, multiple answers were regarded as correct only if all the correct answers were selected. Correctly answered questions contributed 1 point, and incorrectly answered questions contributed 0 points. The crude score of questions related to infectious diseases ranged from 0 to 7, with a higher score indicating higher knowledge of infectious diseases. For the item of willingness to be vaccinated against COVID-19, we assigned 1, 2, 3, 4, and 5 points respectively to the 5 options of very reluctant, reluctant, neutral, willing, and very willing. To comprehensively evaluate the students’ knowledge of infectious diseases, IRT was used to fit the model of 7 items of the questionnaire. Frequently used in studies on education examinations, IRT is a set of psychometric models used to measure unobservable characteristics of the respondents and the development of scoring scales [17-19]. IRT can be used to explain the relationship between a latent trait (eg, the health literacy of school children related to infectious diseases) and observable characteristics and items (eg, questionnaire answers). IRT has at least 3 model specifications. The one parameter logistic model takes item difficulty into account when evaluating individual ability, whereas the two parameter logistic model additionally considers differential discrimination of items [19,20]. In addition to these 2 models, the three-parameter model (TPM) allows the possibility of guessing [19,20]. In this study, a TPM was selected to calculate the IRT score (Table S2 in Multimedia Appendix 1). To score the students’ latent health literacy, we fitted TPM using the R package “ltm: Birnbaum’s three parameter model” to the 7 questions related to the knowledge of infectious diseases [20]. A higher score meant higher health literacy. We plotted the estimated IRT score of questions related to infectious diseases to visualize the students’ performance (Figures S4-S7 in Multimedia Appendix 1). Although not directly related to our main analyses, we also plotted the item characteristic curves, item information curves, and the test information curve to provide some information regarding the difficulty of the test (Figures S8-S10 in Multimedia Appendix 1).

Finally, to summarize categorial sociodemographic characteristics, the correct answer rates of answering the questions, the pre-post changes in the correct answer rates after the intervention, the COVID-19 vaccination rate, the reasons for nonvaccination, and the percentages of the corresponding variables were calculated. We used mean and SD to describe the crude score, the IRT score of questions related to infectious diseases, and the willingness to vaccinate against COVID-19. We used *t* tests to compare the crude score, the IRT score, and the willingness to be vaccinated against COVID-19 across groups. Regarding the willingness to be vaccinated between 2 groups, we also conducted a stratified analysis based on the parents’ sex. Chi-square tests were carried out on the basis of the correct answer rate, the COVID-19 vaccination rate, and the reason for nonvaccination to investigate differences between the 2 groups. The pre-post changes in the correct answer rates after the intervention were compared between study groups, using the *z* test. Furthermore, since we used class as our intervention unit, we also conducted an additional analysis using class as the primary unit of analysis. This was undertaken to ensure that our class-based examination would yield coherent findings as well (Tables S3-S5 in Multimedia Appendix 1). A *P* value less than .05 was considered significant. All data were analyzed using SPSS (version 26; IBM Corp) and R (version 4.2.0; The R Foundation).

### Power

We calculated the power of this study on the basis of the sample size of the intervention and on the primary outcome. To calculate power, we used the sample size of 1862 (intervention group: n=1187; control group: n=675), an acceptable probability for type I error of .05, a pooled SD of 0.767, and a minimal difference in the infectious disease knowledge scores between the 2 groups of 0.166 (ie, *μ*_1_–*μ*_2_). The power of this study was 99.43%.

### Data Exclusion

First, when an intervention group student decided to quit or was lost to follow-up, the student was excluded from the primary analysis. Second, the researchers checked information such as IP address, birth date, sex, and school and class codes to identify duplicates.

## Results

### Study Population

In the baseline survey, 2177 (intervention group: n=1306; control group: n=871) student questionnaires and 2496 (intervention group: n=1430; control group: n=1066) parent questionnaires were collected, amounting to response rates of 86.2% and 98.8%, respectively. In the postintervention survey, 1862 (intervention group: n=1187; control group: n=675) student questionnaires and 1799 (intervention group: n=1076; control group: n=723) parent questionnaires were retrieved, yielding response rates of 73.7% and 71.2%, respectively (Tables S6-S9 in Multimedia Appendix 1). In the intervention group, 2493 (intervention group: n=1306; control group: n=1187) student questionnaires were collected, with a response rate of 95.1%. In the control group, 1546 (baseline survey: n=871; postintervention survey: n=675) student questionnaires were collected, with a response rate of 63.6%.

There were no significant differences in baseline characteristics between the intervention and control groups (Table 1). In the intervention group, there were 691 male and 615 female students; the corresponding numbers in the control group were 459 and 412, respectively. The proportion of households earning less than ¥5000 (US $702.97) was relatively small in both groups (9.8% and 8.9%). Finally, the proportions of students whose parents had college education and above was 72.7% in both groups.

**Table 1:** Sociodemographic characteristics of third-grade students from 11 pilot schools in Longgang District of Shenzhen, China.

Characteristics		Intervention group, n/n (%)	Control group, n/n (%)
**Sex**			
	Male	691/1306 (52.9)	459/871 (52.7)
	Female	615/1306 (47.1)	412/871 (47.3)
**Monthly household income (¥^a^)**			
	<5000	140/1430 (9.8)	95/1066 (8.9)
	5000~10,000	359/1430 (25.1)	267/1066 (25.0)
	10,000~20,000	403/1430 (28.2)	311/1066 (29.2)
	≥20,000	528/1430 (36.9)	393/1066 (36.9)
**Parent’s educational level**			
	Junior high or below	116/1430 (8.1)	82/1066 (7.7)
	High school or technical secondary school	275/1430 (19.2)	209/1066 (19.6)
	College and above	1039/1430 (72.7)	775/1066 (72.7)

^a^¥1=US $0.1445.

### Correct Answer Rates of Questions Related to Infectious Diseases

At baseline, the correct answer rates for questions related to influenza symptoms, influenza preventive measures, and norovirus diarrhea symptoms were different between the intervention and control groups. Specifically, the correct answer rate was higher in the control group (Table 2). In terms of the correct answer rates for questions regarding influenza transmission, chicken pox symptoms, mumps transmission, and COVID-19 symptoms, there were no significant differences between the 2 groups (Table 2). After the intervention, the differences between the 2 groups in the correct answer rates for questions regarding influenza symptoms, influenza preventive measures, and norovirus diarrhea symptoms were no longer observed (Table 2). By contrast, the differences in the correct answer rates for questions regarding chicken pox symptoms, mumps transmission, and COVID-19 symptoms between the 2 groups at the end point were significant, such that intervention group outperformed the control group (Table 2).

**Table 2:** The correct answer rates for questions related to infectious diseases in the intervention and control groups.

Questions		Total, %	Intervention group, %	Control group, %	*P* value
**Baseline**					
	Influenza symptoms	67.57	65.39	70.84	.008
	Influenza transmission	81.86	81.47	82.43	.57
	Influenza preventive measures	84.66	83.08	87.03	.01
	Norovirus diarrhea symptoms	56.41	54.21	59.70	.01
	Chicken pox symptoms	28.34	28.79	27.67	.57
	Mumps transmission	6.89	7.27	6.31	.39
	COVID-19 symptoms	29.54	28.33	31.34	.13
**End point**					
	Influenza symptoms	86.09	86.77	84.89	.26
	Influenza transmission	78.30	78.69	77.63	.60
	Influenza preventive measures	92.91	93.09	92.59	.69
	Norovirus diarrhea symptoms	72.93	73.80	71.41	.26
	Chicken pox symptoms	43.18	47.01	36.44	<.001
	Mumps transmission	10.15	13.23	4.74	<.001
	COVID-19 symptoms	49.14	52.40	43.41	<.001

Regarding the pre-post changes in the correct answer rates after the intervention, the differences between the 2 groups were significant for all items (Table 3). Specifically, the correct answer rates for questions regarding influenza symptoms, influenza preventive measures, norovirus diarrhea symptoms, chicken pox symptoms, and COVID-19 symptoms increased in both groups (for all, *P*<.001). However, the correct answer rates of the intervention group increased more than those of the control group. In the intervention group, the correct answer rate for questions regarding mumps transmission increased in the intervention group but decreased slightly in the control group. Compared with that before the intervention, the correct answer rate for questions regarding influenza transmission decreased slightly after the intervention (Table 3).

**Table 3:** Pre-post changes in the correct answer rates after the intervention in the intervention and control groups.

Questions	Total, %	Intervention group, %	Control group, %	*P* value
Influenza symptoms	18.52	21.38	14.05	<.001
Influenza transmission	–3.56	–2.78	–4.80	.02
Influenza preventive measures	8.25	10.01	5.56	<.001
Norovirus diarrhea symptoms	16.52	19.59	11.71	<.001
Chicken pox symptoms	14.84	18.22	8.77	<.001
Mumps transmission	3.26	5.96	–1.57	<.001
COVID-19 symptoms	19.60	24.07	12.07	<.001

### Crude and IRT Scores for Questions Related to Infectious Diseases

Before the intervention, there was a significant difference in the mean scores for questions regarding infectious disease knowledge between the 2 groups. The mean IRT score of the intervention group (–0.0375, SD 0.7784) was significantly lower (*P*=.01) than that of the control group (0.0477, SD 0.7481). After the intervention, the mean IRT score of the intervention group (0.0543, SD 0.7569) surpassed that of the control group (–0.1115, SD 0.7307). Notably, the postintervention mean score of the intervention group increased from that at baseline, whereas the control group displayed an opposite trend (Table 4). The situation is similar for the crude score (Table 4).

**Table 4:** The crude and item response theory (IRT) score of questions related to infectious diseases in the intervention and control groups.

	Crude score, mean (SD)				IRT-based score, mean (SD)		
	Intervention group	Control group	*P* value	Intervention group		Control group	*P* value
Baseline	3.49 (1.628)	3.65 (1.552)	.02	–0.0375 (0.7784)		0.0477 (0.7481)	.01
End point	4.45 (1.469)	4.11 (1.420)	<.001	0.0543 (0.7569)		–0.1115 (0.7307)	<.001

### COVID-19 Vaccination Rates

The COVID-19 vaccination rates of the intervention and the control groups at baseline were 94.8% and 93.2%, respectively; by the end of the program, they increased slightly to 97.6% and 96.6%, respectively. The differences, however, were not significant (Table 5).

**Table 5:** The COVID-19 vaccination rates of third-grade students before and after the intervention.

	Intervention group, n/n (%)	Control group, n/n (%)	*P* value
Baseline	1238/1306 (94.8)	812/871 (93.2)	.13
End point	1158/1187 (97.6)	652/675 (96.6)	.23

### Willingness to Get Vaccinated and the Reasons for Not Being Vaccinated

Among the study participants who have not been vaccinated against COVID-19, the differences between students’ and parents’ willingness to receive the vaccine in the 2 groups were not significant (Table S10 in Multimedia Appendix 1). After stratifying by parents’ sex, the differences between the 2 groups were still not significant (Table S11 in Multimedia Appendix 1). For students who had not been vaccinated against COVID-19 after the intervention, the students and their parents were worried about side effects among many other reasons (Table S12 in Multimedia Appendix 1).

## Discussion

### Principal Results

Using a quasi-randomized controlled design, this study assessed the effectiveness of a video-assisted health education program sequenced by peer education on infectious disease health literacy among school students. The results suggest that the proposed multicomponent model of health education improved the knowledge of infectious diseases among students, and are consistent with those of previous randomized controlled trials in health education among primary school students [12,21,22]. Moreover, this study not only showcases an innovative approach to raise awareness of disease prevention by incorporating technology and behavioral elements, but also represents a preliminary effort to test the effectiveness of an infectious disease health education program using IRT-based scores.

Our results encapsulate important implications for the practice of health education and healthy behavior promotion. First, the inexpensive and convenient innovative health education approach proposed in this study represents a viable approach to improve student health literacy during pandemics and should be considered in future programs of healthy behavior promotion among school students. The fact that the program was effective among third-grade students does not restrict the potential of this approach since senior students are likely to capture the contents of the program better than third-grade students. Second, the results from the second session of this study partially indicate that web-based teaching may also be an effective tool to promote student engagement in health education, which has been highlighted in previous studies but not confirmed [7].

The possible long-term effects of the first session from our findings should not be ignored. The postintervention survey was carried out immediately after the second education session (including chicken pox, mumps, and COVID-19) and 4 months after the first education session (including influenza and norovirus diarrhea). Despite the time elapsed, the correct answer rates of questions related to infectious diseases that were of focus in the first session were still higher in the intervention group than in the control group. Therefore, third-grade primary school students may endure the impact of health education for at least 4 months. Given the low likelihood of frequently setting up health education sessions in schools, the slow waning of the program’s effects is a desirable feature. However, the cross-over effect from the second session could not be ruled out. For example, the learning of COVID-19 may strengthen the students’ previous understanding of influenza and increase the effect of intervention in influenza. In addition, the second session may sensitize the students in the intervention group. They may review the knowledge of the first session to prepare for the postintervention quiz, which may also enhance the effect of the first session.

It is noteworthy that there was some difference in response rates between the interventional and control groups. The difference in response rates might be attributable to an absence of treatment blinding. In fact, the intervention in this study could not be blinded due to its physical nature, in which case, the intervention group students might be motivated by the education sessions to meet the expectation of the educators to respond to the surveys.

In addition, there was no significant difference in the correct answer rate for questions related to flu transmission routes before or after the intervention, but the pre-post changes in the correct answer rates was different between the 2 groups, and the intervention group performed better than the control group. Owing to countrywide vaccination campaigns, the COVID-19 vaccination rates between the intervention and control groups were not significantly different. The results of the 2 questionnaire surveys showed that the vaccination rates of the 2 groups increased, which was related to the local epidemic and the country's policy encouragement for vaccination.

### Limitations

Several limitations of the study should be noted when interpreting the results. First, we did not collect data on the incidence of related infectious diseases before and after the intervention. A previous study reported that in areas with a high incidence of infectious disease, the health education package had no overall effect in preventing infections. However, the intervention was effective in preventing infections in areas where the baseline prevalence was relatively low [21]. Further studies are needed to explore the impact of our composite intervention on preventing infections. Second, this study was limited in its ability to evaluate component-specific versus composite effects of the educational video, the didactic lessons, the cooperative learning exercises, and peer engagement. The 2-arm trial design could not parse out the influence of each element. Future work should incorporate multiple comparison arms to better isolate the impacts of intervention components. Third, we regret that we did not measure changes in attitudes and behaviors after the intervention, as the health education package is hypothesized to influence these aspects. This is a gap that exists in our study, which future research could explore. Fourth, we used a self-rating questionnaire to collect data. Although self-reporting is a common and accepted method, we could not completely rule out the possibility of measurement error. However, the reliability and validity of self-reporting among children aged >8 years have been shown to be good in health-related questionnaires [23,24]. Fifth, the contamination in this study may underestimate the effect of our intervention. We adopted a clustered quasi-randomized controlled trial design to mitigate within-class person-to-person contamination, although interclass contamination caused by students and teachers could not be eliminated. However, the contamination, if any, happened more likely to the first session rather than the second session since students were physically isolated during the latter. Sixth, as we did not receive the questionnaire from the students lost to follow-up, the primary analysis was not intent-to-treat. Seventh, the second session of health education originally scheduled to enter the campus was changed to web-based classes owing to the serious local epidemic. Therefore, the students were required to fill in the web-based questionnaire at home, which affected the independence of the participants in answering questions; hence, the correct answer rates of the 2 groups were generally higher than those at baseline. Besides, the recovery of the questionnaire was decreased probably due to the lack of the teachers’ supervision outside the schools. However, the missing rates were balanced between the 2 groups, thereby reducing the chances of influencing our conclusions. Moreover, the effect of the health education provided herein may be underestimated because this missing group of students and parents might have lower health literacy, in which case, the intervention would have incremental value.

### Comparison With Prior Work

Despite these limitations, the primary strengths of our study are that it is the first quasi-randomized controlled trial to evaluate the effect of a video-assisted health education program sequenced by peer education on the health literacy of COVID-19 and other infectious diseases among school children, and it is also the first to report IRT scores for questions related to the infectious diseases. Additionally, while our study is quasi-randomized, the allocation process likely achieved reasonable randomization, effectively balancing confounding factors across study arms as evidenced by the systematic allocation of students to intervention or control groups based on their odd or even class numbers, as outlined in Table 1. Importantly, the allocation of students to odd or even classes was not based on systematically different characteristics, as the Ministry of Education of the People’s Republic of China does not permit students to be segregated into different classes based on specific attributes. Therefore, the grouping of students based on class number parity can be considered to approximate the effects of randomization. Moreover, the sample size in this study allowed minimal chances of underpowered analyses. Previous studies might have engaged nonrandomized designs such that mixed results were reported [12,21,22,25-30]. Although most studies demonstrated that the health intervention is effective in improving health knowledge and health literacy, a quasi-randomized controlled trial in China found that the intervention’s effect was not significant among primary school students [25]. Moreover, a number of studies adopted self-control, or observational designs, based on which solid conclusions are difficult to derive [3-5,26-30].

### Conclusions

Our study confirmed that the combination of video-assisted and peer education in a health education program had significant effects on school children. In addition, the effect of the first health education session may endure after 4 months. As such, the proposed program was effective in improving health literacy related to infectious diseases among school children and should be considered for en masse health promotion campaigns during pandemics.

## Appendix

Multimedia Appendix 1 Supplementary figures and tables.

Multimedia Appendix 2 CONSORT eHEALTH checklist (V 1.6.1).

## Abbreviations

IRT: item response theory
TPM: three-parameter model

## Data Availability

The data presented in this study are not publicly available because of ethical requirements but are accessible upon reasonable request to the corresponding author.
