# Supplementary material for: The Effect of a Video-Assisted Health Education Program Followed by Peer Education on the Health Literacy of COVID-19 and Other Infectious Diseases Among School Children: Quasi-Randomized Controlled Trial
Source: JMIR Hum Factors. 2024 Jan 29;11:e43943. doi: 10.2196/43943 (PMC10862245; doi:10.2196/43943)
Supplement: Multimedia Appendix 1 [file humanfactors_v11i1e43943_app1.docx]

**FigureS1** Trial Registration with Longgang District Bureau of Health


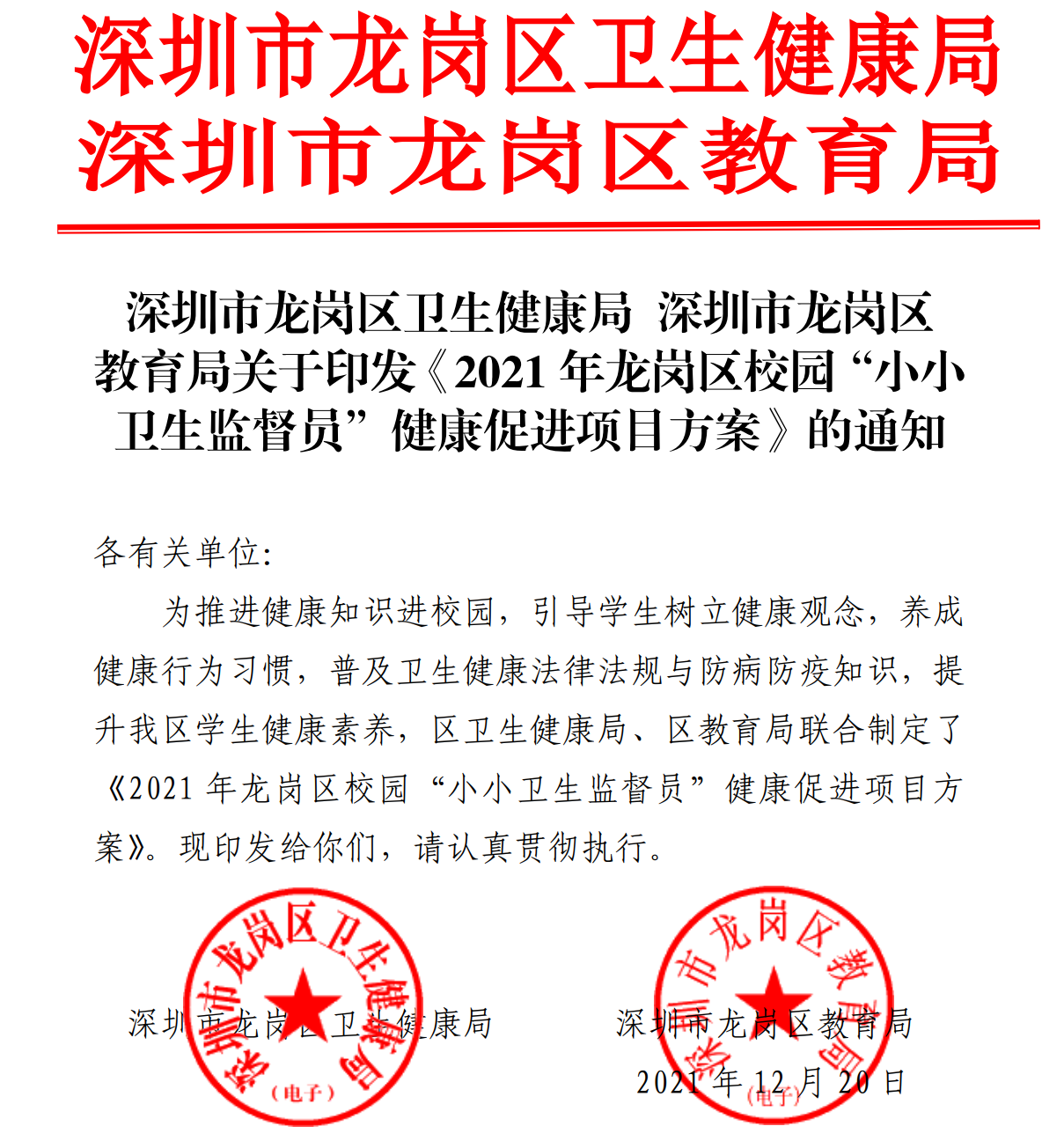


**Intervention Development**

As a public service project launched by district government, the intervention was developed by both researchers and local health department, building on a pilot survey and the Theory of Planned Behavior [13]. First, according to the annual infectious disease surveillance report from the local Bureau of Health, a group of school seasonal infectious diseases was selected. Second, a pilot survey was conducted in two classes (101 students) from one school, which focused on the infectious disease knowledge, comprehension ability, and length of attention among school students. The school teachers also provided information on student’s characteristics and what kind of the health education they needed. Third, working with infectious disease specialist from local Centers for Disease Control and Prevention, a set of intervention measurements were finally developed. Fourth, the intervention designs were further refined throughout communication with implementation personnel (health unit staff) to make it feasible. This approach to intervention development was consistent with the Patient Centered Outcomes Research Institute (PCORI) recommendation for developing interventions to increase participant recruitment and retention, improve outcomes, and increase research validity and relevance to the real world [14].

For the first session, one professionally trained teacher was assigned to each school. The teacher played the finished courseware and video clips through PowerPoint (Figure S2). During the lecture, there would be interactive question and answer sections to incentivize learning. For the second session, the health education had to be conducted in the form of recorded course videos due to a local COVID-19 outbreak (Figure S2). The details of the intervention system are listed in the following table:

**Table S1** Intervention content on third-grade students of 11 pilot schools from December 2021 to April 2022

| Items | Actions to take | Information and content conveyed | Theory of Planned Behavior Factor |
| --- | --- | --- | --- |
| **The first session** |  |  |  |
| 1. Lecture: norovirus diarrhea and influenza | Listen to the teacher | Transmission, symptoms, prevention and control of norovirus and influenza | Utility, risk perception, knowledge, social benefit, experience, cues to action |
| 1. A 5-minute science video: norovirus diarrhea and influenza | Watch a video | Transmission, symptoms, prevention and control of norovirus and influenza | Utility, risk perception, knowledge, social benefit, experience, cues to action |
| 1. Lecture and video: hand hygiene | Listen to the teacher  Watch a video  Practice in class | When should we wash our hands, the proper steps and precautions for washing hands | Utility, knowledge, social benefit, experience, context determinant (access, cues to action) |
| 1. Interactive question and answer sections | Raise hand  Answer the question  Get a little gift | The questions related to norovirus diarrhea and influenza | Utility, risk perception, knowledge, experience, social benefit, cues to action |
| 1. Electing five little health supervisors^a^ | Pick up brochures, armbands and stickers (five little health supervisors) | Supervising the learning of health knowledge and the development of good hygiene habits of their classmates | Utility, knowledge, attitude, experience, context determinant (access, cues to action), perceived behavioral control, plus: peer education in article |
| 1. Encouraging dissemination of knowledge | Take health knowledge to the people around them | The dissemination of knowledge | Utility, knowledge, attitude, social benefit, perceived behavioral control, plus: the two-step flow theory in article |
| **The second session** |  |  |  |
| 1. Recorded course video: chickenpox, mumps and COVID-19 | Watch a video | Pathogens, transmission, symptoms, prevention and control of chickenpox, mumps and COVID-19 | Utility, risk perception, knowledge, social benefit, experience, cues to action |
| 1. A 5-minute science video: chickenpox, mumps and COVID-19 | Watch a video | Pathogens, transmission, symptoms, prevention and control of chickenpox, mumps and COVID-19 | Utility, risk perception, knowledge, social benefit, experience, cues to action |
| 1. Exercises to consolidate | Think and answer | The questions related to chickenpox, mumps and COVID-19 | Utility, risk perception, knowledge, social benefit, experience, cues to action |
| 1. Encouraging dissemination of knowledge | Take health knowledge to the people around them | The dissemination of knowledge | Utility, knowledge, attitude, social benefit, perceived behavioral control, plus: the two-step flow theory in article |

^a^ The mean age was 9 years old, and the ratio of male to female was about 1:1.

**FigureS2** The teacher played the finished courseware and video clips

**
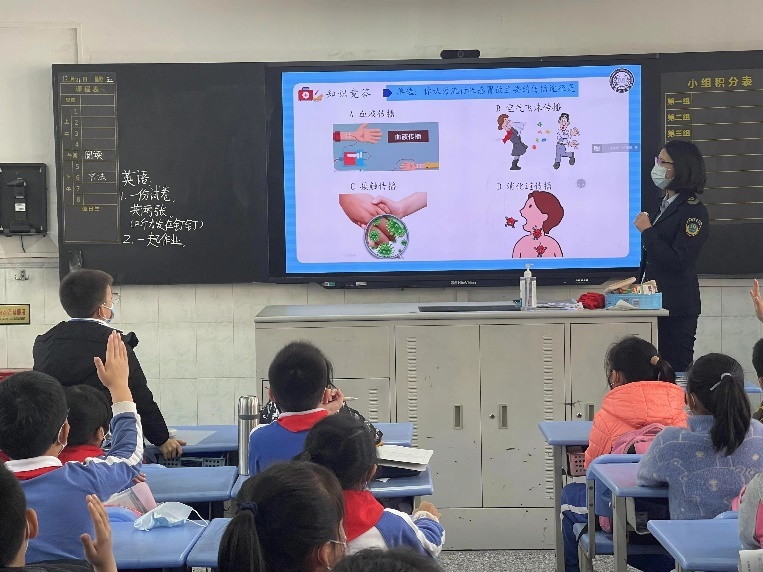
**

**FigureS3** Students watched the recorded course videos at home

**
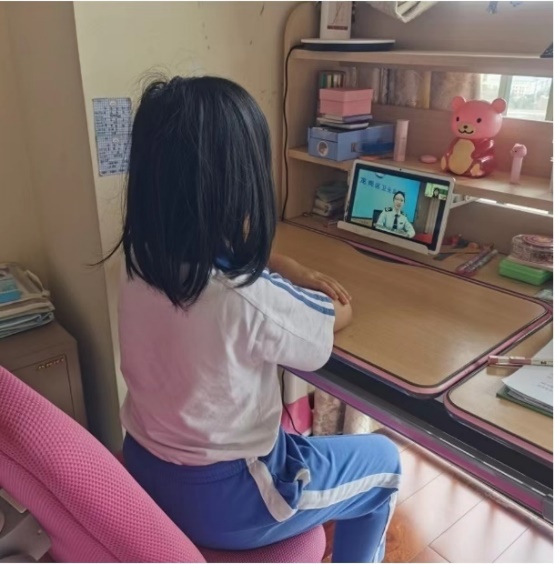
**

**Table S2** IRT model parameters

| Model | -2LL^a^ | AIC ^b^ | BIC ^c^ |
| --- | --- | --- | --- |
|  |  |  |  |
| **Rasch** | 14912.78 | 14928.77 | 14974.26 |
| **TPM** | 14236.76 | 14278.77 | 14398.16 |

^a^ LL: Log-Likelihood.

^b^ AIC: Akaike Information Criterion.

^c^ BIC: Bayesian Information Criterion.

**Figure S4** Baseline IRT score of questions related to infectious diseases in the intervention group


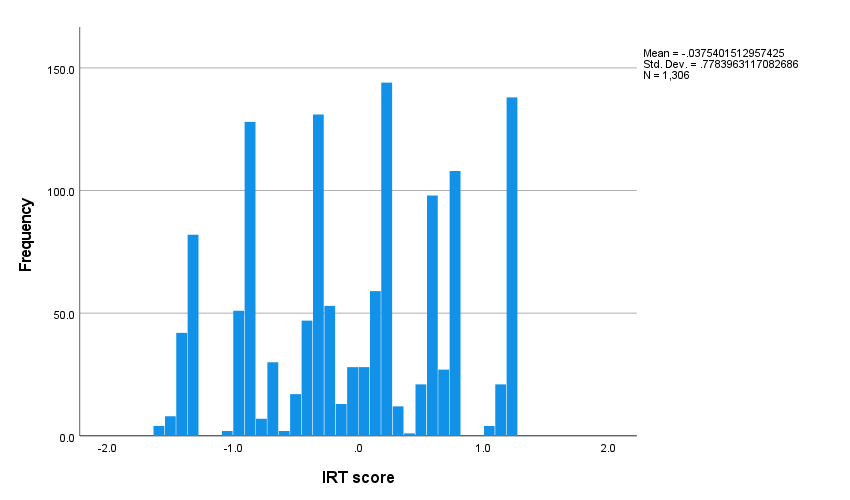


**Figure S5** Baseline IRT score of questions related to infectious diseases in the control group


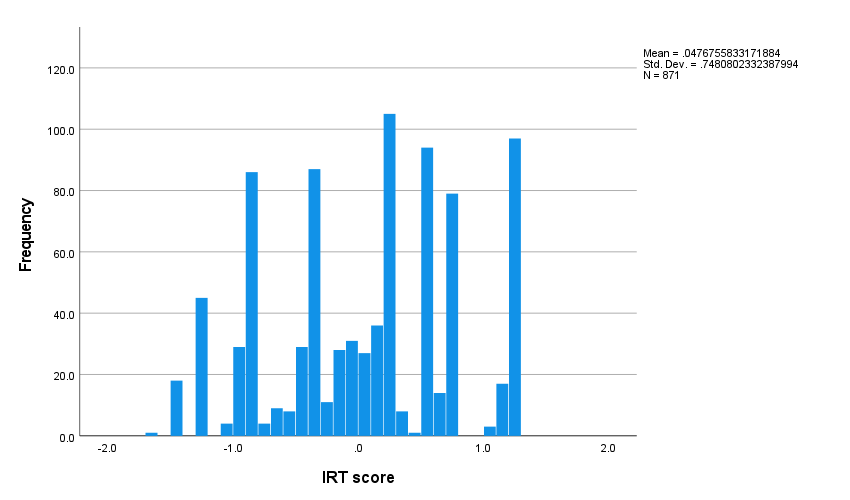


**Figure S6** Post-intervention IRT score of questions related to infectious diseases in the intervention group


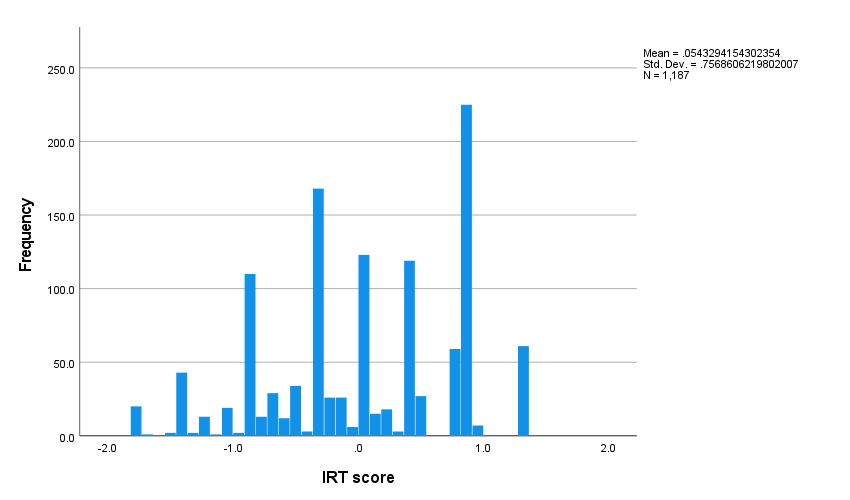


**Figure S7** Post-intervention IRT score of questions related to infectious diseases in the control group


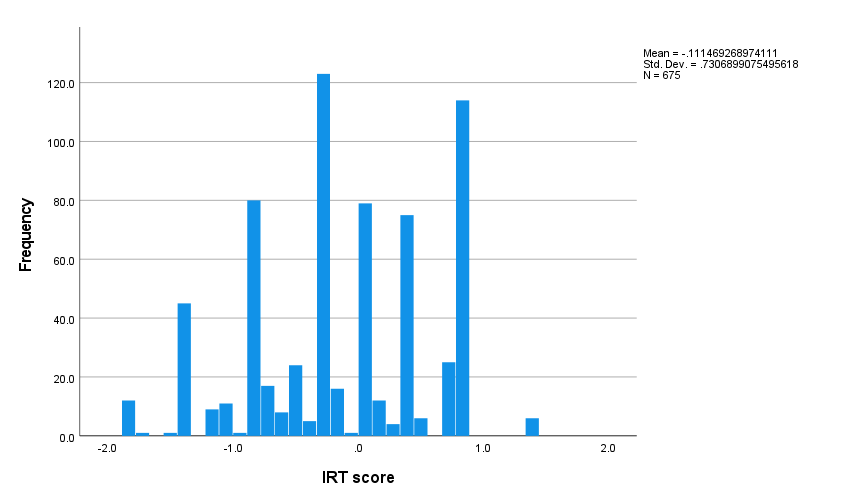


**Figure S8** Item characteristic curves of the seven questions related to infectious diseases


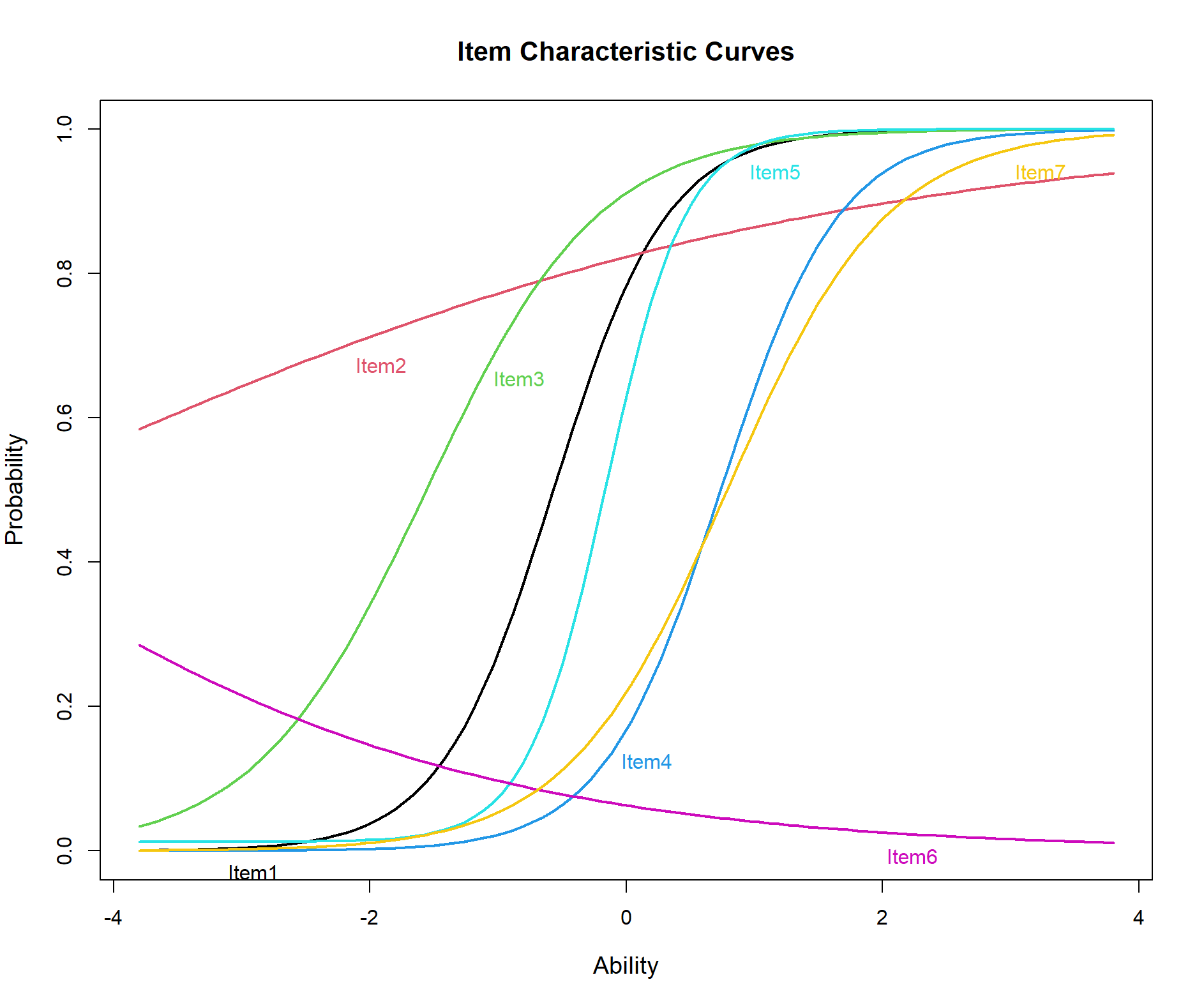


**Figure S9** Item information curses of the seven questions related to infectious diseases


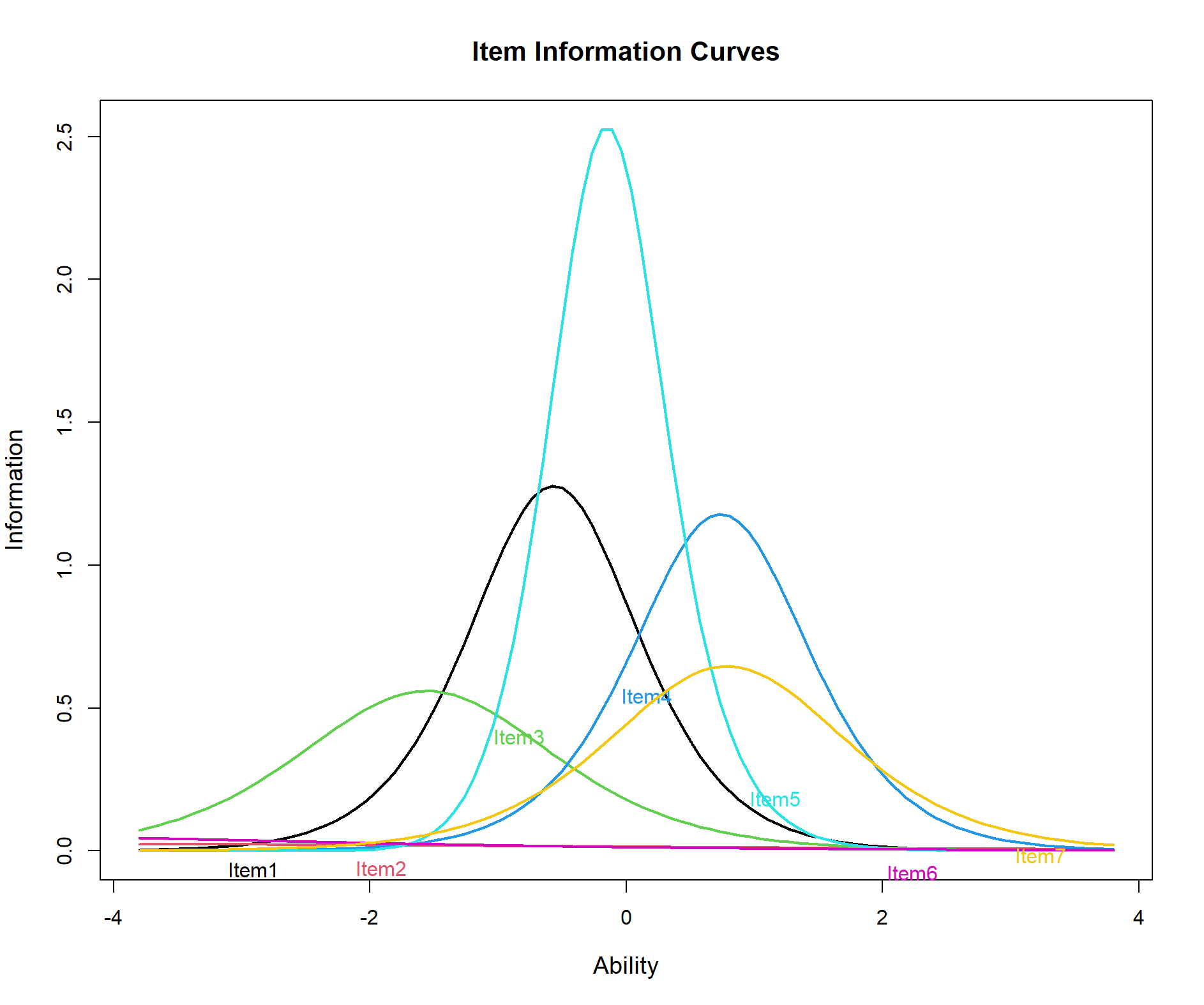


**Figure S10** Test information function of the seven questions related to infectious diseases


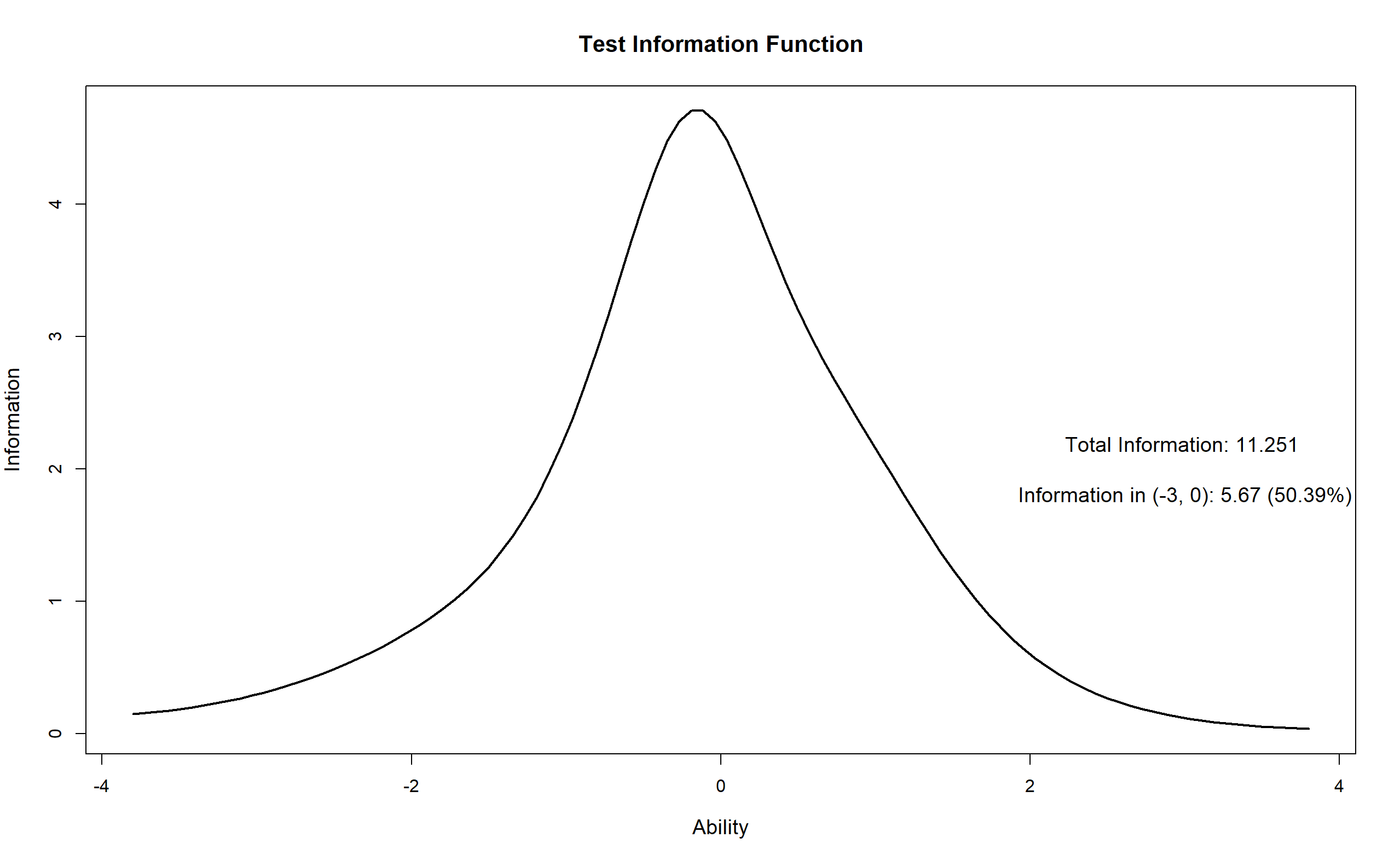


**Table S3** The correct answer rates of questions related to infectious diseases in the intervention and control group (class as the unit of analysis)

| Questions | Total  (%) | Intervention group(%) | Control group(%) | *P* |
| --- | --- | --- | --- | --- |
|  |  |  |  |  |
| **Baseline** |  |  |  |  |
| Influenza symptoms | 67.45 | 63.15 | 72.31 | ＜.001 |
| Influenza transmission | 81.89 | 81.70 | 82.10 | .83 |
| Influenza preventive measures | 84.66 | 82.29 | 87.34 | .004 |
| Norovirus diarrhea symptoms | 56.36 | 52.17 | 61.10 | ＜.001 |
| Chickenpox symptoms | 28.64 | 28.30 | 29.03 | .74 |
| Mumps transmission | 7.13 | 7.42 | 6.81 | .62 |
| COVID-19 symptoms | 29.41 | 27.42 | 31.66 | .05 |
| **Endpoint** |  |  |  |  |
| Influenza symptoms | 85.42 | 86.92 | 83.46 | .04 |
| Influenza transmission | 79.44 | 80.44 | 78.13 | .23 |
| Influenza preventive measures | 92.48 | 93.38 | 91.30 | .10 |
| Norovirus diarrhea symptoms | 71.69 | 73.78 | 68.96 | .03 |
| Chickenpox symptoms | 42.22 | 48.05 | 34.64 | ＜.001 |
| Mumps transmission | 10.74 | 14.67 | 5.63 | ＜.001 |
| COVID-19 symptoms | 47.62 | 53.15 | 40.43 | ＜.001 |

**Table S4** Pre-post changes in the correct answer rates after the intervention in the intervention and control group (class as the unit of analysis)

| Questions | Total  (%) | Intervention group(%) | Control group(%) | *P* |
| --- | --- | --- | --- | --- |
|  |  |  |  |  |
| Influenza symptoms | 17.97 | 23.77 | 11.15 | ＜.001 |
| Influenza transmission | -2.45 | -1.26 | -3.97 | ＜.001 |
| Influenza preventive measures | 7.82 | 11.09 | 3.96 | ＜.001 |
| Norovirus diarrhea symptoms | 15.33 | 21.61 | 7.86 | ＜.001 |
| Chickenpox symptoms | 13.58 | 19.75 | 5.61 | ＜.001 |
| Mumps transmission | 3.61 | 7.25 | -1.18 | ＜.001 |
| COVID-19 symptoms | 18.21 | 25.73 | 8.77 | ＜.001 |

**Table S5** The crude and IRT score of questions related to infectious diseases in the intervention and control group (class as the unit of analysis)

|  | Crude score, mean (SD) | | | IRT-based score, mean (SD) | | |
| --- | --- | --- | --- | --- | --- | --- |
|  | Intervention group | Control group | *P* | Intervention group | Control  group | *P* |
|  |  |  |  |  |  |  |
| **Baseline** | 3.43 (0.903) | 3.70 (0.717) | .24 | -0.0712 (0.4537) | 0.0735 (0.3654) | .23 |
| **Endpoint** | 4.50 (0.304) | 4.03 (0.435) | <.001 | 0.0692 (0.1588) | -0.1650 (0.2410) | <.001 |

**Table S6** Baseline survey of the students in the intervention group and control group in December 2021

| Characteristics | Intervention group(N=1306) | | Control group (N=871) | | |
| --- | --- | --- | --- | --- | --- |
|  | n | % | n | | % |
|  |  |  |  | |  |
| **Sex** |  |  |  | |  |
| Male | 691 | 52.9 | 459 | | 52.7 |
| Female | 615 | 47.1 | 412 | | 47.3 |
| **Influenza symptoms^a^** |  |  |  | |  |
| Fever | 1120 | 85.8 | 773 | | 88.7 |
| Fatigue | 1052 | 80.6 | 714 | | 82.0 |
| Headache | 1023 | 78.3 | 701 | | 80.5 |
| Cough | 1187 | 90.9 | 824 | | 94.6 |
| **Influenza transmission** |  |  |  | |  |
| Blood transmission | 25 | 1.9 | 14 | | 1.6 |
| Air droplet transmission | 1064 | 81.5 | 718 | | 82.4 |
| Insect-borne transmission | 61 | 4.7 | 41 | | 4.7 |
| Digestive tract transmission | 156 | 11.9 | 98 | | 11.3 |
| **Influenza preventive measures^a^** |  |  |  | |  |
| Getting the flu shot | 1212 | 92.8 | 820 | | 94.1 |
| Washing hands frequently | 1206 | 92.3 | 808 | | 92.8 |
| Keeping indoor ventilation | 1192 | 91.3 | 813 | | 93.3 |
| Wearing a mask when going out in public | 1225 | 93.8 | 831 | | 95.4 |
| **Chickenpox symptoms^a^** |  |  |  | |  |
| Fever | 833 | 63.8 | 576 | | 66.1 |
| Headache | 539 | 41.3 | 345 | | 39.6 |
| Nausea | 735 | 56.3 | 451 | | 51.8 |
| Skin papules and blisters all over the body | 1213 | 92.9 | 822 | | 94.4 |
| **Norovirus diarrhea symptoms^a^** |  |  |  | |  |
| Diarrhea | 1074 | 82.2 | 748 | | 85.9 |
| Nausea | 1131 | 86.6 | 775 | | 89.0 |
| Stomachache | 977 | 74.8 | 703 | | 80.7 |
| Fever | 999 | 76.5 | 663 | | 76.1 |
| **Mumps transmission^a^** |  |  |  | |  |
| Air droplet transmission | 1143 | 87.5 | 783 | | 89.9 |
| Digestive tract transmission | 652 | 49.9 | 438 | | 50.3 |
| Mother-to-child transmission | 409 | 31.3 | 258 | | 29.6 |
| Insect-borne transmission | 365 | 27.9 | 213 | | 24.5 |
| **COVID-19 symptoms^a^** |  |  |  | |  |
| Fever | 1197 | 91.7 | 816 | | 93.7 |
| Dry cough | 1138 | 87.1 | 770 | | 88.4 |
| Fatigue | 1075 | 82.3 | 741 | | 85.1 |
| Stuffy nose | 910 | 69.7 | 589 | | 67.6 |
| Runny nose | 904 | 69.2 | 630 | | 72.3 |
| Sore throat | 968 | 74.1 | 668 | | 76.7 |
| Decreased sense of smell and taste | 781 | 59.8 | 539 | | 61.9 |
| [Conjunctivitis](javascript:;) | 560 | 42.9 | 397 | | 45.6 |
| Muscle pain | 759 | 58.1 | 541 | | 62.1 |
| Diarrhea | 752 | 57.6 | 506 | | 58.1 |
| **COVID-19 vaccination** |  |  |  | |  |
| Having been vaccinated | 1238 | 94.8 | 812 | | 93.2 |
| Not yet vaccinated | 68 | 5.2 | 59 | | 6.8 |
| **Reason for not vaccinating** |  |  |  | |  |
| Thinking you won't get infected | 5 | 7.4(5/68) | 5 | | 8.5(5/59) |
| Worrying about side effects | 20 | 29.4(20/68) | 23 | | 39.0(23/59) |
| Considering the vaccine is ineffective | 2 | 2.9(2/68） | 1 | | 1.7(1/59) |
| Others | 41 | 60.3(41/68) | 30 | | 50.8(30/59) |
| **Willingness to vaccinate now** |  |  |  | |  |
| Very willing | 17 | 25.0(17/68) | | 22 | 37.3(22/59) |
| Willing | 18 | 26.5(18/68) | | 13 | 22.0(13/59) |
| Neutral | 20 | 29.4(20/68) | | 11 | 18.6(11/59) |
| Reluctant | 7 | 10.3(7/68) | | 11 | 18.6(11/59) |
| Very reluctant | 6 | 8.8(6/68) | | 2 | 3.4(2/59) |

^a^multiple-answers question

**Table S7** Baseline survey of the parents in the intervention group and control group in December 2021

| Characteristics | Intervention group(N=1430) | | | Control group(N=1066) | | |
| --- | --- | --- | --- | --- | --- | --- |
|  | n | | % | n | % | |
|  |  | |  |  |  | |
| **Parent’s sex** |  | |  |  |  | |
| Male | 462 | | 32.3 | 356 | 33.4 | |
| Female | 968 | | 67.7 | 710 | 66.6 | |
| **Monthly household income** |  | |  |  |  | |
| <5000 | 140 | | 9.8 | 95 | 8.9 | |
| 5000~10000 | 359 | | 25.1 | 267 | 25.0 | |
| 10000~20000 | 403 | | 28.2 | 311 | 29.2 | |
| ≥20000 | 528 | | 36.9 | 393 | 36.9 | |
| **Parent’s educational level** |  | |  |  |  | |
| Junior high or below | 116 | | 8.1 | 82 | 7.7 | |
| High school or technical secondary school | 275 | | 19.2 | 209 | 19.6 | |
| College and above  or above | 1039 | | 72.7 | 775 | 72.7 | |
| **COVID-19 vaccination** |  | |  |  |  | |
| Having been vaccinated | 1358 | | 95 | 1000 | 93.8 | |
| Not yet vaccinated | 72 | | 5.0 | 66 | 6.2 | |
| **Reason for not vaccinating** |  | |  |  |  | |
| Thinking the child won't get infected | 0 | | 0.0(0/72) | 0 | 0.0(0/66) | |
| Worrying about side effects | 14 | | 19.4(14/72) | 15 | 22.7(15/66) | |
| Considering the vaccine is ineffective | 1 | 1.4(1/72) | | 1 | | 1.5(1/66) |
| Others | 57 | 79.2(57/72) | | 50 | | 75.8(50/66) |
| **Willingness to vaccinate your child now** |  |  | |  | |  |
| Very willing | 17 | 23.6(17/72) | | 16 | | 24.2(16/66) |
| Willing | 19 | 26.4(19/72) | | 11 | | 16.7(11/66) |
| Neutral | 11 | 15.3(11/72) | | 15 | | 22.7(15/66) |
| Reluctant | 19 | 26.4(19/72) | | 16 | | 24.2(16/66) |
| Very reluctant | 6 | 8.3(6/72) | | 8 | | 12.1(8/66) |

**Table S8** Post-intervention survey of the students in April 2022

| Characteristics | Intervention group(N=1187) | | Control group(N=675) | |
| --- | --- | --- | --- | --- |
|  | n | % | n | % |
|  |  |  |  |  |
| **Sex** |  |  |  |  |
| Male | 633 | 53.3 | 357 | 52.9 |
| Female | 554 | 46.7 | 318 | 47.1 |
| **Influenza symptoms^a^** |  |  |  |  |
| Fever | 1137 | 95.8 | 632 | 93.6 |
| Fatigue | 1117 | 94.1 | 623 | 92.3 |
| Headache | 1104 | 93.0 | 617 | 91.4 |
| Cough | 1140 | 96.0 | 647 | 95.9 |
| **Influenza transmission** |  |  |  |  |
| Blood transmission | 12 | 1.0 | 7 | 1.0 |
| Air droplet transmission | 934 | 78.7 | 524 | 77.6 |
| Insect-borne transmission | 67 | 5.6 | 40 | 5.9 |
| Digestive tract transmission | 174 | 14.7 | 104 | 15.4 |
| **Influenza preventive measures^a^** |  |  |  |  |
| Getting the flu shot | 1158 | 97.6 | 651 | 96.4 |
| Washing hands frequently | 1161 | 97.8 | 663 | 98.2 |
| Keeping indoor ventilation | 1156 | 97.4 | 661 | 97.9 |
| Wearing a mask when going out in public | 1149 | 96.8 | 651 | 96.4 |
| **Chickenpox symptoms^a^** |  |  |  |  |
| Fever | 1006 | 84.8 | 531 | 78.7 |
| Headache | 666 | 56.1 | 329 | 48.7 |
| Nausea | 744 | 62.7 | 374 | 55.4 |
| Skin papules and blisters  all over the body | 1167 | 98.3 | 661 | 97.9 |
| **Norovirus diarrhea symptoms^a^** |  |  |  |  |
| Diarrhea | 1131 | 95.3 | 630 | 93.3 |
| Nausea | 1125 | 94.8 | 621 | 92.0 |
| Stomachache | 1020 | 85.9 | 584 | 86.5 |
| Fever | 1011 | 85.2 | 567 | 84.0 |
| **Mumps transmission^a^** |  |  |  |  |
| Air droplet transmission | 1111 | 93.6 | 620 | 91.9 |
| Digestive tract transmission | 796 | 67.1 | 502 | 74.4 |
| Mother-to-child transmission | 511 | 43.0 | 205 | 30.4 |
| Insect-borne transmission | 411 | 34.6 | 242 | 35.9 |
| **COVID-19 symptoms^a^** |  |  |  |  |
| Fever | 1170 | 98.6 | 660 | 97.8 |
| Dry cough | 1145 | 96.5 | 644 | 95.4 |
| Fatigue | 1123 | 94.6 | 642 | 95.1 |
| Stuffy nose | 992 | 83.6 | 544 | 80.6 |
| Runny nose | 986 | 83.1 | 535 | 79.3 |
| Sore throat | 1099 | 92.6 | 621 | 92.0 |
| Decreased sense of smell and taste | 1021 | 86.0 | 567 | 84.0 |
| [Conjunctivitis](javascript:;) | 752 | 63.4 | 392 | 58.1 |
| Muscle pain | 931 | 78.4 | 478 | 70.8 |
| Diarrhea | 856 | 72.1 | 462 | 68.4 |
| **COVID-19 vaccination** |  |  |  |  |
| Having been vaccinated | 1158 | 97.6 | 652 | 96.6 |
| Not yet vaccinated | 29 | 2.4 | 23 | 3.4 |
| **Reason for not vaccinating** |  |  |  |  |
| Thinking you won't get infected | 1 | 3.4(1/29) | 4 | 17.4(4/23) |
| Worrying about side effects | 9 | 31.0(10/29) | 3 | 13.0(3/23) |
| Considering the vaccine is ineffective | 0 | 0.0(0/29) | 1 | 4.3(1/23) |
| Others | 19 | 65.5(19/29) | 15 | 65.2(15/23) |
| **Willingness to vaccinate now** |  |  |  |  |
| Very willing | 6 | 20.7(6/29) | 10 | 43.5(10/23) |
| Willing | 9 | 31.0(9/29) | 5 | 21.7(5/23) |
| Neutral | 5 | 17.2(5/29) | 3 | 13.0(3/23) |
| Reluctant | 6 | 20.7(6/29) | 3 | 13.0(3/23) |
| Very reluctant | 3 | 10.3(3/29) | 2 | 8.7(2/23) |

^a^multiple-answers question

**Table S9** Post-intervention survey of the parents in April 2022

| Characteristics | Intervention group(N=1076) | | Control group(N=723) | |
| --- | --- | --- | --- | --- |
|  | n | % | n | % |
|  |  |  |  |  |
| **Parent’s sex** |  |  |  |  |
| Male | 277 | 25.7 | 209 | 28.6 |
| Female | 799 | 74.3 | 523 | 71.4 |
| **Monthly household income** |  |  |  |  |
| <5000 | 128 | 11.9 | 75 | 10.2 |
| 5000~10000 | 296 | 27.5 | 190 | 26.0 |
| 10000~20000 | 265 | 24.6 | 202 | 27.6 |
| ≥20000 | 387 | 36.0 | 265 | 36.2 |
| **Parent’s educational level** |  |  |  |  |
| Junior high or below | 100 | 9.3 | 43 | 5.9 |
| High school or technical secondary school | 224 | 20.8 | 152 | 20.8 |
| College and above  or above | 752 | 69.9 | 537 | 73.4 |
| **COVID-19 vaccination** |  |  |  |  |
| Having been vaccinated | 1051 | 97.7 | 711 | 97.1 |
| Not yet vaccinated | 25 | 2.3 | 21 | 2.9 |
| **Reason for not vaccinating** |  |  |  |  |
| Thinking the child won't get infected | 0 | 0.0(0/25) | 0 | 0.0(0/21) |
| Worrying about side effects | 7 | 28.0(7/25) | 8 | 38.1(8/21) |
| Considering the vaccine is ineffective | 2 | 8.0(2/25) | 1 | 4.8(1/21) |
| Others | 16 | 64.0(16/25) | 12 | 57.1(12/21) |
| **Willingness to vaccinate now** |  |  |  |  |
| Very willing | 8 | 32.0(8/25) | 6 | 28.6(6/21) |
| Willing | 3 | 12.0(3/25) | 4 | 19.0(4/21) |
| Neutral | 5 | 20.0(5/25) | 2 | 9.5(2/21) |
| Reluctant | 7 | 28.0(7/25) | 5 | 23.8(5/21) |
| Very reluctant | 2 | 8.0(2/25) | 4 | 19.0(4/21) |

**The willingness to vaccinate and the reasons for not being vaccinated**

Among the research subjects who have not been vaccinated against COVID-19, the students' willingnesses to be vaccinated in the intervention group and the control group before the intervention were 3.49(SD 1.228) and 3.71(SD 1.246), respectively, and the parents' willingnesses to let their children receive the COVID-19 vaccine were 3.31(SD 1.318) and 3.17(SD 1.365), respectively. After the intervention, the students' willingnesses to be vaccinated in the intervention group and control group were 3.31(SD 1.318) and 3.78(SD 1.380), respectively, and the parents' willingnesses to let their children receive the COVID-19 vaccine were 3.32(SD 1.406) and 3.14(SD 1.558), respectively. The differences were not statistically significant (Table S10).

**Table S10** The willingnesses to vaccinate among student and parent questionnaire

|  | Intervention group,  mean (SD) | Control group,  mean (SD) | *P* |
| --- | --- | --- | --- |
|  |  |  |  |
| **Student questionnaire** |  |  |  |
| Baseline | 3.49(1.228) | 3.71(1.246) | .31 |
| Endpoint | 3.31(1.312) | 3.78(1.380) | .21 |
| **Parent questionnaire^a^** |  |  |  |
| Baseline | 3.31(1.318) | 3.17(1.365) | .54 |
| Endpoint | 3.32(1.406) | 3.14(1.558) | .69 |

^a^In the parent questionnaire, the question is “how willing you are to have your child vaccinated against COVID-19 right now”

For the parent questionnaire, we further explored whether the differences in the willingness to vaccinate between the two groups were changed across parent’s sex. After stratified by parent’s sex, the fathers' willingnesses to be vaccinated in the intervention group and the control group before the intervention were 3.41(SD 1.260) and 3.28(SD 1.487), respectively, and the mothers' willingnesses to let their children receive the COVID-19 vaccine were 3.26(SD 1.352) and 3.10(SD 1.300), respectively. After the intervention, the fathers' willingnesses to be vaccinated in the intervention group and control group were 3.25(SD 1.753) and 2.60(SD 1.517), respectively, and the mothers' willingnesses to let their children receive the COVID-19 vaccine were 3.35(SD 1.272) and 3.31(SD 1.580), respectively. The differences were not statistically significant (Table S11).

**Table S11** The willingnesses to vaccinate among parent questionnaire stratified by parent’s sex

|  | Intervention group,  mean (SD) | Control group,  mean (SD) | *P* |
| --- | --- | --- | --- |
|  |  |  |  |
| **Male** |  |  |  |
| Baseline | 3.41(1.260) | 3.28(1.487) | .75 |
| Endpoint | 3.25(1.753) | 2.60(1.517) | .51 |
| **Female** |  |  |  |
| Baseline | 3.26(1.352) | 3.10(1.300) | .56 |
| Endpoint | 3.35(1.272) | 3.31(1.580) | .94 |

The reasons for not being vaccinated were analyzed for 52 students and their parents who had not been vaccinated against the COVID-19 vaccine in the survey after the intervention. Worrying about side effects and many other reasons were the main reasons why students were reluctant to receive the vaccine or parents were reluctant to let their children receive it. Most of the other reasons filled in in detail were contraindications for vaccination, such as previous allergic reactions or acute diseases and some were personal choices.

**Table S12** The reasons for not being vaccinated among student and parent questionnaire

| Item | Intervention group(%) | Control group(%) | *P* |
| --- | --- | --- | --- |
|  |  |  |  |
| **Students** |  |  |  |
| I don't think I can get infected | 3.4(1/29) | 17.4(4/23) | .11 |
| I'm worried about the side effects | 31.0(10/29) | 13.0(3/23) |  |
| I don't think the vaccine is effective | 0.0(0/29) | 4.3(1/23) |  |
| Others^a^ | 65.5(19/29) | 65.2(15/23) |  |
| **Parents** |  |  |  |
| I don't think my child will get infected | 0.0(0/25) | 0.0(0/21) | .81 |
| I'm worried about the side effects | 28.0(7/25) | 38.1(8/21) |  |
| I don't think the vaccine is effective | 8.0(2/25) | 4.8(1/21) |  |
| Others^a^ | 64.0(16/25) | 57.1(12/21) |  |

^a^For those who choose this item, we will pop up the blank and ask the subject to fill in the specific reason.
